# SECONDARY AMPUTATION: A QUALITATIVE STUDY OF QUALITY OF LIFE IN PATIENTS AFTER PRIMARY LIMB SALVAGE SURGERY AND AFTER LATER ABLATIVE SARCOMA TREATMENT

**DOI:** 10.2340/jrm.v57.34888

**Published:** 2025-01-28

**Authors:** Veronika VETCHY, Carmen TROST, Reinhard WINDHAGER, Gerhard M. HOBUSCH

**Affiliations:** Department of Orthopedics, Medical University of Vienna, Vienna, Austria

**Keywords:** qualitative, quality of life, secondary amputation, patients’ view

## Abstract

**Objective:**

This study focuses on how patients experience the time following amputation after primary limb salvage surgery due to musculoskeletal malignancies. Limb salvage is state of the art in the treatment of musculoskeletal tumours. Nonetheless, in some cases, limb salvage can become problematic over time, resulting in poorer limb function and septic outcomes. This raises the question of whether amputation is indicated sooner rather than later. Patients who have undergone secondary amputation might retrospectively prefer a different approach.

**Design:**

Interview study.

**Subjects/patients:**

Patients who underwent primary limb salvage surgery followed by later ablative sarcoma treatment.

**Methods:**

Semi-structured interviews and the standardized “Prosthetic Limb Users Survey of Mobility” questionnaire were conducted. Interviews were analysed according to Mayring content analysis method.

**Results:**

Amputation is perceived as an improvement after a long course of illness with little quality of life. By enhancing the amputation environment and providing detailed information regarding quality of life afterwards, emotional pressure could be reduced and patient satisfaction improved.

**Conclusion:**

Surgical options for ablation should be openly communicated earlier when consulting patients experiencing recurrent complications that might eventually lead to amputation. Supporting factors that subsequently may help to improve quality of life after amputation were further identified.

## LAY ABSTRACT

This study explores the experiences of patients who have undergone limb reconstruction surgery for lower limb sarcoma and subsequently required amputation. The study focuses on determining the optimal timing for amputation and potential improvements to the procedure. Interviews with patients revealed a preference for a different initial approach. The study indicates that amputation is seen as an improvement after a long struggle with the disease and reduced quality of life. Patients expressed a desire for earlier information concerning the possibility of amputation and detailed information on post-amputation quality of life. The results emphasize the importance of openly communicating surgical alternatives earlier in the treatment process and identifying factors that promote improved quality of life after amputation.

While the WHO clearly defines quality of life (QoL) as “an individual’s perception of their position in life in the context of the culture and value systems in which they live and in relation to their goals, expectations, standards, and concerns”, it is acknowledged that QoL is a broad concept influenced by many different factors (1). While a clear definition of QoL in the literature has not yet been established, some use the term to refer to a patient’s health or physical status, while most consider it as a description of a patient’s perception of QoL influenced by non-medical aspects (2).

At a percentage of 1–2% of adult cancer and an incidence of 6 cases per 100,000, sarcomas are a rare disease. They comprise a broad group of neoplasms of mesenchymal origin with more than 80 histological subtypes (3–5). Multidisciplinary treatment has been considered the preferred approach for about 40 years. Depending on tumour progression and location, the main goal has been to salvage the limb, which includes a wide tumour resection and an eventual reconstruction with grafts or endoprosthetics. However, amputation can sometimes become necessary (6, 7). The treatment can have far-reaching psychological and functional effects on patients’ everyday lives. The decision to amputate must be made cautiously to minimize the impact on the QoL (8).

Amputation can be performed immediately (primary) or delayed (secondary) after alternative measures have been tried (9). Limb salvage is state of the art treatment for musculoskeletal tumours (10). However, in the long term, limb salvage is often associated with numerous complications that may worsen limb function (11). Secondary amputation is often the last option following hospitalization, surgeries, and difficult recovery. Frustration and disappointment can follow the exhausting course of the disease, especially with additional loss of functionality compared with primary amputation. Analysing experiences made after salvage surgery could help patients come to terms with possible amputation (12). Having had secondary amputation, patients might retrospectively choose differently in the first place or at least after a few surgical re-treatments.

## METHODS

The study was conducted in accordance with the Declaration of Helsinki and was approved by the ethics committee of the Medical University of Vienna (EC no 1266/2019). Data were obtained through quantitative and qualitative measures. Therefore, triangulation was used to compare results from different methodological approaches. Triangulation describes the process of combining and integrating findings from quantitative and qualitative components of research. In this case, the qualitative data from semi-structured interviews and the quantitative data from the standardized questionnaire Prosthetic Limb Users Survey of Mobility 12 (Plus M_12), as well as the Visual Analog Scale (VAS), were combined.

### Sample

A sample of 30 patients was chosen consecutively to participate in this study. Due to the long time since amputation, contact information for 8 patients was outdated, and they could not be reached by telephone or post. The goals and methodology of the study, as well as its use as a doctoral thesis by 1 of the authors, were explained beforehand. Four patients never responded, 2 had moved too far away, 1 was diseased, 2 did not want to participate because they did not want to deal with the subject anymore, and 1 declined offering a reason. One patient did not attend the scheduled interview. Nine patients agreed to participate. However, 2 patients were subsequently excluded because their diagnoses did not fit into the final inclusion criteria (1e patient had a non-sarcoma diagnosis, and 1 an upper extremity amputation). Instead, 2 patients who met the inclusion criteria and had regular appointments at the Department of Orthopedics agreed to participate in the interview study spontaneously.

Participants in the study included 4 male and 5 female sarcoma patients with a median age of 54 years (range 22 to 65 years) who had secondary amputations of the lower extremities at different levels (Table I).

**Table I:** Sample characteristics

Sample characteristics (*n* = 9)	*n* (%)
Gender	
Female	5 (56)
Male	4 (44)
Age range	22–65 (Median: 54)
Family status	
Married/with partner	7 (78)
Single	2 (22)
Children	6 (67)
Employment	
Full time	2 (22)
Part time	2 (22)
Rent	1 (11)
Unemployed	2 (22)
Early retirement	2 (22)
Student	2 (22)
Income	
Below 1,000 euros	2 (22)
Between 1,000 and 2,000 euros	4 (44)
Between 2,000 and 3,000 euros	2 (22)
Above 3,000 euros	1 (11)
Amputation height	
Hip disarticulation	2 (22)
Transfemoral	5 (56)
Knee disarticulation	1 (11)
Forefoot	1 (11)
Time since amputation	5 weeks to 18 years (median: 2 years)

The inclusion criteria for this study were survivors of sarcoma who underwent 2 or more limb salvage procedures and then had secondary amputation. Patients had to be registered and treated at the Medical University of Vienna, Department of Orthopedics and Trauma Surgery. The patients included in this study constitute a portion of the sample from the RDA (Research, Documentation, and Analysis) 1364/2018 register, an automatically generated data pool of routine clinical data of the Medical University of Vienna (13). On the other hand, they are also part of the “1412/2018 patient-reported outcome measurements after orthopedic surgery as a basis for prediction of functioning in patients with sarcoma and total joint replacement sarcoma-research (PROMSAR) Endoprosthetic-Research (PROMENDOR)” study. The data were not duplicated due to the qualitative study design. The study was approved by the ethics committee of the Medical University of Vienna (EC no 1266/2019).

### Quantitative measurement

The PLUS-M 12 was used to determine the mobility of patients. To measure the level of pain, the VAS was used to document what the patients were then experiencing. VAS is a scale that allows patients to rate their subjective pain by noting it on a visual 10 cm beam, with 0 being no pain and 10 being the most pain imaginable. The position of the note can be measured, and the individual pain level can be translated to a number between 0 and 10. In the PLUS M12 Test, 12 questions are asked, and patients can give 1 (unable to do) to 5 (without any difficulty) points, with a maximum of 60 points. To show how the scores correlate with the amount of sport they engage in daily, the sports activity was sorted into 3 categories. The first category, “various sports activities” was assigned to patients who described themselves as participating in many different kinds of sports activities regularly. The second category, “some sport”, describes patients who engaged in less frequent and less strenuous activities. The third category, “no sport”, includes patients who do not participate in any kind of sport activity.

### Qualitative measurement

**Fig. 1:**
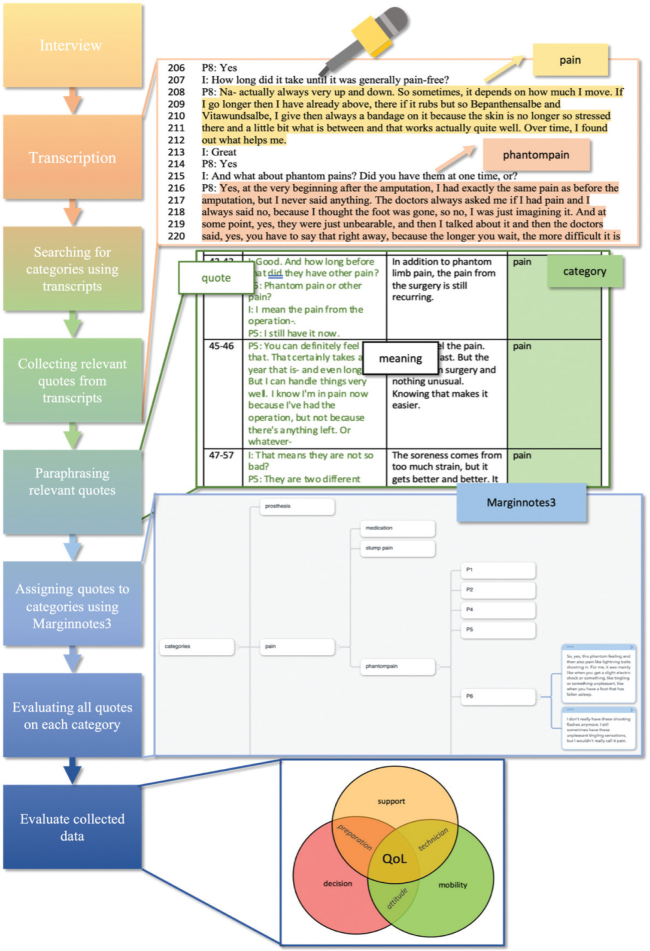
Qualitative measurement: how Mayring content analysis is conducted using first the transcribed interviews to create categories and later sorting them by categories and subcategories using Marginnote 3. Copyright © 2020 Beijing YunSi Software. This example shows how the category “pain” and subcategories like “phantom pain” are found, how transcripts are assessed using those categories, and how found data are combined and evaluated.

Additive qualitative data were collected through semi-structured interviews, which lasted an average of 31 min (ranging from 17–50 min, Fig. 1).

Semi-structured interviews allow for open questions in a structured setting. This prepared setting provides comparable qualitative data while open questions enable a deeper understanding of the topic (14). The interviews were conducted in German by 2 authors: 1 was a medical student at the University of Vienna and the other was a researcher experienced in qualitative research with a master’s degree in sociology (VV and CT). The interviews took place at the Department of Orthopedic Surgery at the Medical University of Vienna. Some patients chose to have family members present at the interview.

**Fig. 2:**
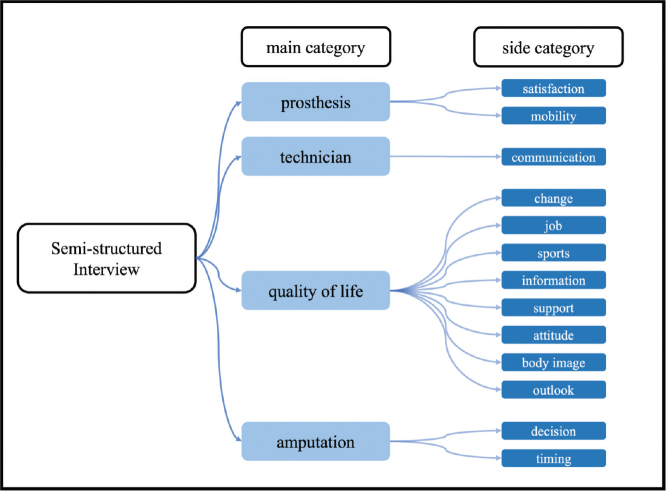
Main categories and side categories developed referring to Mayring.

**Fig. 3:**
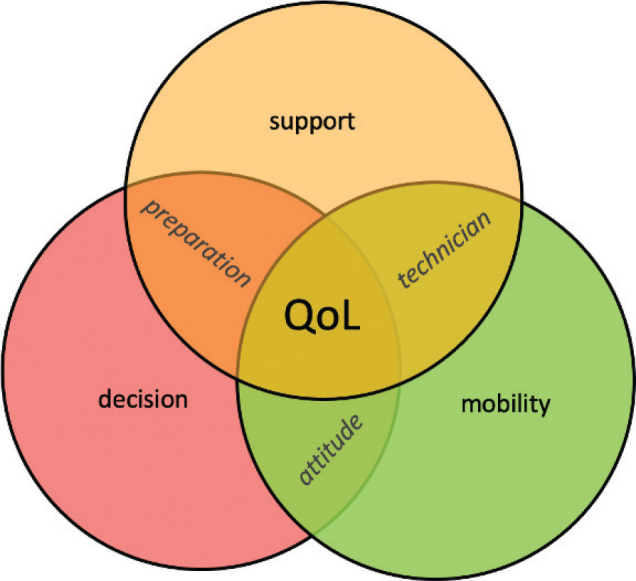
Overlapping influences on QoL.

The interview guide, structured into 4 parts, included questions concerning prosthesis satisfaction, pain, the impact of amputation on the patient’s everyday life, and sociodemographic questions, e.g., “How have you experienced this life change so far?”. Although the interview was based on these questions, the participants were encouraged to introduce and elaborate on topics they considered important. The semi-structured interviews were recorded and later transcribed. The analysis used the mind-mapping tool MarginNote 3 (https://www.marginnote.com/). The interviews were evaluated using the Mayring content analysis method: main categories (prosthesis, orthopaedic technician, pain, QoL, amputation) and side categories were formulated inductively (Fig. 2) based on detailed participant accounts and evaluated after that. The Mayring content analysis method adopts the following principles. Participants’ statements (Fig. 3) were organized and analysed using the developed categories. With repeated review and revision by all authors of the categories and transcripts, the aim was to achieve the highest possible degree of reliability. Qualitative research serves to propose hypotheses concerning correlations that can then be tested with the help of further quantitative research (15). We used the Consolidated Criteria for Reporting Qualitative Research checklist to ensure comprehensive reporting of our study.

## RESULTS

Amputation influences the patient’s life in many ways (see Fig. 1). The following describes the 5 main categories developed according to Mayring (Table SI).

### Prosthesis

Seven of the 9 participating patients wear a prosthesis, relying heavily on it to assist them in their daily activities.

*Satisfaction.* For most, it took some time until the prosthesis worked satisfactorily. Some patients have still not found solutions for problems like socket fitting. P8 decided to change prostheses after experiencing inflammation due to a poor fit with the first one and is now much happier. However, she explains that not being able to wear the prosthesis during the inflammation period took a significant toll on her mood. The abilities a prosthesis provides are very important to her QoL.

Two patients do not wear a prosthesis. P11 has not yet received one because the amputation was recent and postoperative changes are still ongoing. P2 decided against wearing one due to recurring blistering.

*Mobility.* All patients who have a prosthesis said that they wear it all day, except when staying at home. In such cases, other aids like chairs with wheels in the kitchen are used to replace the prosthesis.

Due to a various movements, the prosthesis is exposed to diverse loads, which can lead to various problems like blistering or tenderness.

Another limitation is that some patients experience issues with the prosthesis if they sit for long periods.

### Orthopaedic technician

A good relationship with the orthopaedic technician is crucial for prosthesis satisfaction. Four of the interviewees did not stay with their first orthopaedic technician, mainly due to poor communication or dissatisfaction with the resulting prosthesis. Patients expect extensive technical knowledge from their technicians. Each patient has diverse needs, and it means a lot to them when technicians go out of their way to find the best prosthesis.

*Communication.* Establishing good communication with the technician is vital for amputee satisfaction. It is important for patients to feel that the technician listens to their individual problems and takes them seriously. However, because amputation can be a sensitive issue, patients might not like discussing this. One patient explained that she only felt comfortable when she found a technician who is also an amputee himself and therefore understands the problems firsthand.

P2: He knows what it’s all about because it affects him, too. And that’s who I’m with now and that’s where it feels right. You can tell him that something hurts or something like that and I don’t like that or that would be an idea.… He taught me everything.

### Quality of life

The day-to-day life of all patients was greatly influenced by their amputation. Loss of mobility and flexibility in time management imposes many different challenges that patients must overcome.

*Change.* QoL changes after amputation were described from 2 perspectives. For most, the journey is divided into life with an endoprosthesis before infection and then the time with worsening infection and gradual loss of function. Compared with the time before infection, amputation was seen as a loss of function and mobility by most. However, compared with the time during infection, most see amputation as an improvement.

P1: I was already so very limited in my mobility. The ankle joint was already stiffened at the bottom because, during the operation cement had gone into the ankle joint. The infection was not really under control; I needed antibiotics again and again, and the danger that something is there, that something becomes septic again, or … that is no longer the case. I don’t have a foreign body in there. I can do many things that I could not do before.

Life after amputation is very different from before. All patients describe that their everyday life runs more slowly. Everything has to be planned more precisely. New strategies have to be developed for many activities.

*Job.* The impact on patients’ QoL of not being able to work should not be underestimated. Patients who were working at the time stopped working upon diagnosis or during the period of recurring infections. Two are on early retirement, and 2 others are still unemployed. One is on early retirement not just because of the amputation but because of the combination of chemotherapy, the amputation, and an unrelated hernia. Another stopped working after amputation and is currently unsure if he wants to continue working. For 4 patients, working is a possibility. For those who had to stop working, amputation was a way back to a daily paid work routine. Now, 4 of the 9 patients are working. Two of them even manage to study and work simultaneously.

*Sports.* The type of sports that are still perceived as possible after the amputation varies (Table II). All participants are quite active in their daily lives. Even those who said they are not doing any sports use home training tools and take care of their household. Most, however, report being active. Two of the participants do not wear a prosthesis at the moment, which influences the types of sports they can participate in. The patient who decided against finding a new prosthesis also does not enjoy any kind of sports. One is still in the process of getting a prosthesis and is looking forward to trying out the possibilities. Many sports require a great deal of personal initiative to practise. Especially to ride a bicycle again, many individual techniques were found. For most people, mobility returns slowly and requires a lot of training. Despite this, most do not let themselves lose hope. Insurmountable limits are drawn differently. The only limitation on which all interviewees agreed was that running is no longer possible.

**Table II:** Overview: category sports

ID	Sports	Type	No longer possible	Category	Prosthesis in use
1	Yes	Skiing, swimming, tennis, hiking	–	3 (various)	Yes
2	No	–	Motorcycling	1 (none)	No
4	Yes	Walking	–	2 (some)	Yes
5	Yes	Triathlon, snowboarding, mountaineering, cycling	Running, spontaneity	3 (various)	Yes
6	No	–	Running, dancing	1 (none)	Yes
7	Yes	Swimming,	Kayaking, cycling	2 (some)	Yes
8	Yes	cycling, trampoline, gymnastics,	Running, gymnastics club	3 (various)	Yes
9	No	Swimming	Cycling, running, hiking	1 (none)	Yes
11	Not yet	Billiards, hopefully in the future	ball sports	1 (none)	No

Table indicates which sports the patients can and cannot do and to which category/amount of sports they are assigned.

Swimming is a sport that is relatively easy to master, both with and without a prosthesis. All sports require a slow approach, where the psyche is as much of an obstacle as the motoric limitation.

*Information.* Patients’ sources of information differ greatly. One is a healthcare provider himself, 1 has a family member who works in the healthcare system and can provide information, and another already knew someone who had had an amputation and relied on them. Five patients reported that they had received very little information from healthcare professionals at the hospital. Many believed that they had received most of the information from the rehab centre professionals, where contacts that were established with other patients there played a major role. In general, other affected people are an important source of information, but it is not easy to find them, especially for the younger patients. Most people would appreciate a contact point for information and networking.

P8: For a long time I was looking for someone to share experiences with. I have acquaintances who are a bit older [incomprehensible], but you must have someone to exchange.

*Support.* For all interviewees, support and acceptance by their families and friends were very important. If there were other affected individuals or people with expertise in their immediate environment, this provided additional support. Experiences with rehabilitation varied widely. Some patients were very satisfied, while others reported various problems. Rehabilitation services were not always equipped to address individual issues. One patient mentioned that due to the rules for choosing rehabilitation in Austria, patients were on average significantly older and had different problems than she did and the caregivers were not equipped to address her specific needs.

*Attitude.* Eight of the nine interviewees had a very positive mindset. The consistent attitude of not questioning the situation and avoiding depression was a recurring theme in interviews. One individual struggled significantly with her current situation, partly because she had set her goals too high and lost her positive attitude due to repeated setbacks. She has also disliked the prosthesis and was using crutches at the time of the interview.

P4: Without a strong will, which you have to have, nothing will work. Nothing. Because when you look at yourself in the mirror. Just, just when you see the prosthesis, that’s it, that’s it, that’s it, and that’s it.

*Body image.* Most participants were uncomfortable with their disability being obvious. For patients with hip disarticulation, this this discomfort was primarily expressed in their efforts to improve their gait pattern. Lower amputations are usually easier to conceal, but patients still struggled with wearing short trousers, among other things.

This reluctance was not only related to public appearances but also influenced other areas such as choosing a partner.

*Outlook.* Some patients mentioned during the interviews a certain fear of how ageing would affect their situation. P2, who did not have a prosthesis at the time, was very afraid of not being able to manage her everyday life in old age or that phantom pain would become stronger.

### Amputation

The final indication for amputation in all cases was an infection and/or the danger of a recurrence of the initial sarcoma. Many factors influenced how patients viewed their amputation in retrospect, and their perception of the situation was influenced by how prepared they felt.

*Decision.* Four of the interviewees had the opportunity to decide on amputation themselves, following doctors’ recommendation. For the others, amputation was necessary to save their lives. All but 1 patient did not regret the decision to undergo amputation. One patient expressed this view but decided against a second, higher amputation even though doctors warned it might cost her life. Most noticeable while talking about this issue was that many participants wished they had been confronted with the possibility of amputation before it became necessary. Those who were able to reflect on the possibility of amputation beforehand also expressed gratitude for the chance to prepare.

*Timing.* Five of the patients had to have amputation under time pressure, while 4 had time to consider the decision. Those who had time to think about it found it helpful. None of the patients interviewed, however, mentioned that they would change the amputation date retrospectively. They did not regret not having an amputation earlier, even if it might have spared them pain.

P6: I never thought about it, or even after the amputation, I never said, `Why didn’t you do it before?` It was just the past for me then.

### Pain/Visual Analog Scale (VAS) PLUS Mobility

**Fig. 4:**
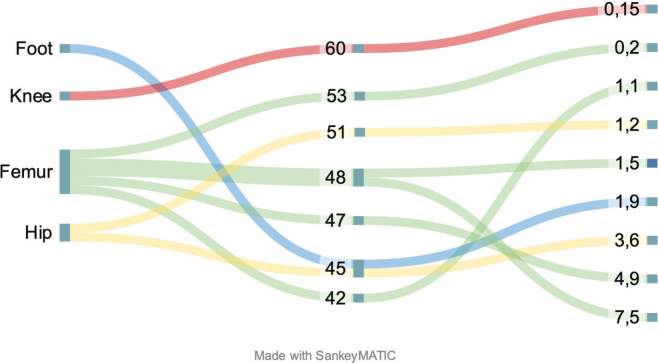
Comparison of patients’ amputation height, PLUS Mobility Score and VAS.

Six out of 9 reported no pain in the residual limb, and the remaining 3 reported pain only after long periods of use. The median PLUS Mobility score was 48 (range 42–60). Although the trend is for high mobility and low pain to correlate, due to the qualitative method and small patient population no quantitative significant correlations could be calculated (Fig. 4).

## DISCUSSION

This study addresses the subjective views of patients who had to undergo secondary amputation on this procedure, their current life situation, and QoL. Due to the limited body of literature on this specific topic, an exploratory, qualitative approach was chosen to highlight the factors that patients perceive as helpful and improve their QoL after secondary amputation.

In recent decades, the quality of life as a measure for patient care and outcomes has become more and more important (16). So far there are only a few studies that deal with the topic of QoL after amputation, and those that do present a wide range of results (17).

Limb-salvage surgery (LSS) is the first choice for the treatment of the lower extremity sarcoma (18). However, there have been studies that suggest the same or better physical and emotional outcomes after amputation. While the question of the differences in psychosocial and functional effects between LSS and primary amputation has already been answered differently (19–21), the data on secondary amputations is even thinner.

Both the cancer and its treatment itself are major disruptions in the lives of patients. Other studies have already pointed to significant changes that fundamentally alter patients` lives (22, 23). This study shows that patients’ mindsets influence how they deal with the new situation. Secondary amputation leads to these changes being joined by additional ones.

Dealing with limb loss is a complex task for those affected, as it involves overcoming both physical and psychological obstacles (24). The adaptation of those affected to the new situation depends on their personal characteristics and psychological resources, as well as the support of family and society (25).

Few qualitative studies highlight important factors affecting QoL in non-dysvascular lower limb amputation (26); however, our study is the first to consider the impact of a cancer diagnosis and subsequent secondary amputation on patients’ psychological well-being. Studies also show that it makes a difference whether the amputation was planned or had to be sudden. Patients who could prepare found the amputation easier to accept (24). This finding supports the results of this study, in which prepared patients described making the decision as particularly important, and others, in retrospect, wished they had this option. It also reinforces the patients’ desire to be confronted with the possibility of amputation earlier in the course of the disease.

The QoL experienced is strongly linked to functionality and mobility in everyday life. Patients in this study described their QoL after amputation as significantly worse than at the time right after LSS when some limb functionality was still preserved. However, they found it significantly improved compared with the time when infections and other defects that later led to amputation had begun. The recurrent infections significantly hindered mobility. Amputation allowed them to resume most activities, but all participants reported limitations affecting both speed and spontaneity. The resumption of sports was particularly important for many. Other studies also show that participation in sports activities positively impacts both physical and mental health (27).

Some studies also support our findings that sports are important for psychological well-being and thus increase the perceived QoL (27–29). All participants reported that movement is an important part of their lives. They described how loss limits and gain of function directly contribute to their psychological well-being. The literature also confirms patients` statements that sports are often practised less and are harder to pursue than before the amputation, due to fewer opportunities and physical limitations (27, 30). Studies have identified various factors influencing patients’ participation in sports, including gender, amputation height, age, and activity before amputation (27, 31, 32).

It is important to note that our findings do not correlate amputation height with less mobility, unlike other studies (33, 34). On the contrary, there is a trend in the opposite direction, which could be attributed to the small sample size and the different age groups of the patients. Although the patients with the highest amputation of the hip showed a strong desire to improve their physical ability, their age ranges were on both ends of the patient sample. This study coincides with previous studies in that younger patients regain more mobility after amputation (35). Most participants report many sports activities before the amputation, following this trend.

The results of this study are consistent with previous ones in that many patients perceived the time gained through LSS as positive and helpful in preparing them for amputation (12). They go even further, however, stating that it is of even greater benefit if the possibility of amputation is actively discussed beforehand.

This study has several limitations. We were not able to generate generalizable statements due to the qualitative nature of our research. However, our study explores patient opinions that may not be fully portrayed by previous research. Validation of our results in cohorts of adequate sample size could provide further information on the relevance of the themes discovered.

Many of the patients who were contacted declined to participate because they did not want to think about the topic anymore. Therefore, it is probable that patients who took part in the interviews either had a better outcome or possessed other qualities that might influence their views on amputation. As the patients included in this study all live in Austria they have a similar ethnic and cultural background, so influencing factors could not be addressed. Additionally, sex- and age-related issues could not be determined. With this qualitative study, we aimed to shed light on the patients’ viewpoints and perceptions and seek to substantiate them by comparing them with other studies.

Amputation is seen as an improvement after a long course of disease with very little QoL. The early mention of the surgical option of ablation during the course of the disease and recurrent complications helps patients cope with the situation if amputation may become necessary.

By improving the circumstances surrounding an amputation, as well as detailed information regarding QoL after the procedure, the emotional pressure can be reduced, thereby influencing patients’ satisfaction. The patients’ willing and independent decision to undergo amputation had a major impact on their subsequent QoL.

The results may help determine the best time to amputate by adding knowledge of psychological influences to the usually considered physical factors to improve long-term QoL.

## Supplementary Material

SECONDARY AMPUTATION: A QUALITATIVE STUDY OF QUALITY OF LIFE IN PATIENTS AFTER PRIMARY LIMB SALVAGE SURGERY AND AFTER LATER ABLATIVE SARCOMA TREATMENT
