# Supplementary material for: SECONDARY AMPUTATION: A QUALITATIVE STUDY OF QUALITY OF LIFE IN PATIENTS AFTER PRIMARY LIMB SALVAGE SURGERY AND AFTER LATER ABLATIVE SARCOMA TREATMENT
Source: J Rehabil Med. 2025 Jan 28;57:34888. doi: 10.2340/jrm.v57.34888 (PMC11795125; doi:10.2340/jrm.v57.34888)
Supplement: SECONDARY AMPUTATION: A QUALITATIVE STUDY OF QUALITY OF LIFE IN PATIENTS AFTER PRIMARY LIMB SALVAGE SURGERY AND AFTER LATER ABLATIVE SARCOMA TREATMENT [file JRM-57-34888-s1.pdf]

Supplementary material has been published as submitted. It has not been copyedited, or typeset by Journal of Rehabilitation Medicine

Table SI. Patient quotes

category	Quotations
Prothesis	P1:“So in the morning at 6 when I get up […] and in the evening - yes partly, if I have a concert in the evening then until half past 11 p.m. so if I'm out somewhere – otherwise […] Exactly the whole day. At home I put the prosthesis down every now and then and use crutches.”<br>P1:"When I cook, I have a stool with wheels, and I ride around on it."<br>Not everyone wants to walk without the crutches at home
Mobility	P5: “ Well, I have bruises from time to time, because I just become more and more active. I do more and more sports with it, go more and more into the mountains or just the safer I feel, I want to try more. Or just because I was snowboarding in winter and then suddenly other strains appear on the socket, because it was no longer a forward and backward strain but more a rotational strain and then I got pressure points on the back.”
Satisfaction	P1: “[…] it actually depends a lot on whether the shaft fits. We had to do a lot of work to find out if it rubs somewhere and if there is pressure on the skin, there will be a problem.”<br>„[…] that was actually in the 10th year after I had the prosthesis for the first time, that I could not put it on for a longer period of time. Yes, and that had a major impact on my mood. I was already in a bad mood, because you can't really do anything. Just lie around “.<br>P8:"Now this is a new system and it's really great because I'm standing on my own thigh and that's just a completely different feeling [...] for the pelvis that you're standing straight again. I can master longer distances again and don't have to put my leg down after each step and wiggle it and then move on to the next step. But now it is much more a smooth movement and I feel much better than before “<br>Orthopedic technician<br>P8: "I just changed the technician a year and a half ago because I was actually dissatisfied for a long time. [..] Now I'm very satisfied again, but at the beginning there was a bit of a confusion, but it had a big effect on my quality of life […] because I was badly taken care of for a long time”.
Communication	P6:" the chemistry must be right. And this is also a rather sensitive area, especially shortly after the amputation. It is all still very painful, somewhere you feel a little embarrassed. It is also an unpleasant feeling, you know you need it, you don't want to admit it, not really.”<br>P2: "I was able to describe it as I needed to. He understood that too. Some people don't even understand what you want”.
Technical knowledge	P8:“because it has actually taken years and they have not been able to match a suitable socket. I don't know what the reason is. [...] In any case, the even the surgical truss maker has already

	told me that he is beginning to doubt his abilities, and this is not very encouraging for me, because I can't do it either".<br>P8:“because it has actually taken years and they have not been able to match a suitable socket. I don't know what the reason is. [...] In any case, the even the surgical truss maker has already told me that he is beginning to doubt his abilities, and this is not very encouraging for me, because I can't do it either".<br>Quality of life
change	P1: “I mean in comparison to how it was before the whole thing, it is of course already a restriction of the quality of life to before the infection.”<br>P1: “Yeah so compared to the time I had the infection [it was an improvement] definitely”<br>P2: “in eight days, it will be two years. [...] B: The worst two years of my life.”<br>“yes the amputation is totally restrictive, so I don't want to gloss over that now. So I try to be very active and I struggle a lot, I struggle to lead the life I led before but it sucks of course. And there is no comparison without a leg, like with a leg, and a lot of things don't work or work but are no fun anymore. And it is already a lot of things an incredible fight. Just until you somehow manage to bite through the pain and frustration, looks from others. So it is already until you have developed the self-confidence or you just dare to try things that is just shit. And that is why amputation is always a major procedure.”
Job	P1: “Yes, that is for example one point. I mean that I can now fully work again - that is of course also for the quality of life - I have been sitting at home for almost a year. That is a factor for quality of life. [...] It's boring up to feeling meaningless or--"
Sports	P9: “I can't ride a bike anymore. I'm afraid I'll fall over”.<br>P8: “Mmmm yes, so I have the pedal in such a way, where I put the toes in. I am now already more secure in cycling and when stopping I know that I can only get down with the other side, that I, because otherwise this can still happen today, if I fall on the wrong side. But now I know that, and it hasn't happened again since”.<br>P4 "I have the (crutches) with me for safety. [...] I don't need it normally, [...] I am not afraid but subconsciously it is there automatically."
Information	P1 : "The time in X in the rehab centre helped me a lot. There you are among like-minded people with those who have been there for a longer time, then you see what is possible, what they do and - ".<br>P9: “Yes, as I said, somehow like that, there is not really a contact point or advice centre about this whole process.”<br>P1:"I have to mention that. My wife and also the children. Yes, that's the way it is now, and fully accepted. But also the support in everyday things makes a big difference”

	P4: "Because, for example, when I get into the shower, I need my husband in the background. I do that alone but that when I slip. I have everything within reach but there must always be someone there. I'll never shower alone."<br>" I could already walk when I went to rehab […] and I wanted to walk better and more beautifully, and they did not know anything about hip-ex patients, because all hip-ex patients they had stayed in a wheelchair."
Attitude	P4: "Yes I will never think negatively about it, never. Because there's no point, there's no point, because then I just ruin everything."
Body image	P8: "I just don't go outside with the crutches, that's just also- […] I am like that. But I just don't like it."<br>P8:"Yes, I have always said I will be alone forever, never find a partner and never do anything with anyone. My parents also wanted to protect me because they said: yes, don't get your hopes too high and I didn't do that either, so - yes and somehow, I don't know how I was allowed to do that, but I was the first in my circle of friends to find a partner and I wasn't even looking for one, it's simply indescribable."
Outlook	"I think I can consider myself really lucky, so anyone who talks to me and says, wow, you don't have phantom pain, you can be happy. I hope it stays that way because I'm kind of afraid that it will become more and more in my old age."<br>"And when I need both hands to vacuum, I just jump. That works now. Once I'm 80, it won't work anymore."
Decision	P5: I think what would have helped me in principle in the whole process would have been if this had been mentioned at some point. I mean by the fact that I still had a long time, I understand that it didn't happen, but I think it would have helped me to hear at some point that amputation can happen at some point".<br>P8: "No, actually, it was all right. I owe that a lot to my mom, because she brought it up about two weeks before the amputation".
Timing	P9: "I went to Doctor X for a check-up appointment and in the end, he suggested that I should consider it. Of course, I still had time to think about it and then one day later I had an appointment. But the timing was about 2, 3 months, I think".<br>"I could discuss it among friends, exactly. But as I said, I also already found the orthopedic technician. I then also took another holiday before that. Exactly".<br>P5: "Well, it wasn't really a process. […] I came to get my last surgery appointment for something else and he said that we have to amputate tomorrow. So, there was no process".
